# Advances in Models of Fibrous Dysplasia/McCune-Albright Syndrome

**DOI:** 10.3389/fendo.2019.00925

**Published:** 2020-01-24

**Authors:** Hsuan Lung, Edward C. Hsiao, Kelly L. Wentworth

**Affiliations:** ^1^Division of Endocrinology and Metabolism and the Institute for Human Genetics, Department of Medicine, University of California, San Francisco, San Francisco, CA, United States; ^2^Oral and Craniofacial Sciences Graduate Program, School of Dentistry, University of California, San Francisco, San Francisco, CA, United States; ^3^Department of Dentistry, Chang Gung Memorial Hospital and College of Medicine, Chang Gung University, Kaohsiung, Taiwan; ^4^Division of Endocrinology and Metabolism, Zuckerberg San Francisco General Hospital, University of California, San Francisco, San Francisco, CA, United States

**Keywords:** GPCR (G protein-coupled receptors), McCune-Albright syndrome (MAS), fibrous dysplasia (FD), GNAS (guanine nucleotide-binding protein/[alpha]-subunit, G_**s**_α, cAMP, mouse models, human cell models

## Abstract

The G_s_ G-protein coupled receptor pathway is a critical regulator of normal bone formation and function. The G_s_ pathway increases intracellular cAMP levels by ultimately acting on adenylate cyclase. McCune-Albright Syndrome (MAS) and fibrous dysplasia (FD) of the bone are two proto-typical conditions that result from increased cellular G_s_ signaling activity. Both are caused by somatic activating mutations in the *GNAS* gene that encodes for the G_s_α subunit. FD bone lesions are particularly difficult to treat because of their variability and because of the lack of effective medical therapies. In this review, we briefly discuss the key clinical presentations of FD/MAS. We also review the current status of mouse models that target the G_s_ GPCR signaling pathway and human cellular models for FD/MAS. These powerful tools and our improving clinical knowledge will allow further elucidation of the roles of GPCR signaling in FD/MS pathogenesis, and facilitate the development of novel therapies for these medically significant conditions.

## Introduction

Musculoskeletal disorders such as skeletal dysplasias are a significant health problem affecting both children and adults. A variety of cellular pathways, including G-protein coupled receptors (GPCRs) and their ligands, have been identified as key regulators of osteoblast formation and function—two critical steps in normal bone formation and homeostasis. The human GPCR family includes over 340 non-olfactory and 400 olfactory receptors (1, 2), making it the largest class of receptors in the human genome. GPCRs mediate a wide variety of biological processes and are activated by multiple types of extracellular signals, ranging from photons and ions to small molecules to peptides. The diversity of GPCRs and their responses to small molecules has made them major targets for over 40% of modern pharmaceuticals (3).

GPCRs signal through a select number of canonical pathways (4): among these, the G_s_ and G_i_ pathways increase or decrease intracellular cAMP levels, respectively, by acting on adenylate cyclase, while the G_q_ pathway increases intracellular calcium by activating phospholipase C.

**Figure 1:**
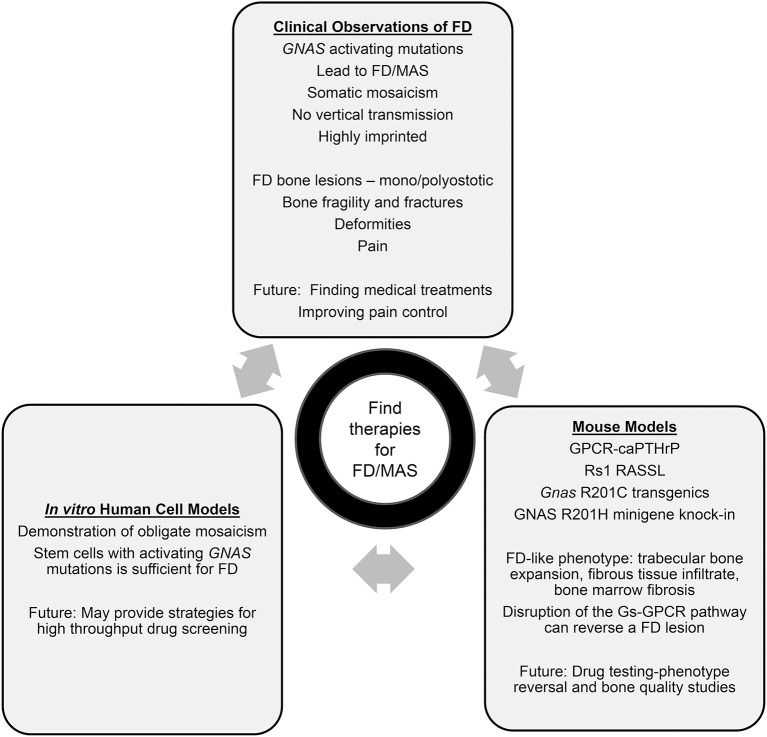
Summary of human, mouse, and clinical findings in FD.

McCune-Albright Syndrome (MAS) is a proto-typical disease caused by activating mutations in the *GNAS* locus, encoding the G_s_α protein (5). MAS is a mosaic genetic disease characterized by the classic triad of polyostotic fibrous dysplasia (FD) of the bone, café-au-lait skin hyperpigmentation, and peripheral precocious puberty. Patients with MAS may have other endocrinopathies, including Cushing's syndrome, hyperthyroidism, acromegaly, and solid organ malignancies of the breast, thyroid, and pancreas. FD/MAS is caused by an acquired somatic mutation in *GNAS*, the gene that encodes the alpha subunit of the stimulatory G-protein (G_s_α), leading to constitutive activation of G_s_ signaling in affected cells. This mutation occurs post-zygotically, resulting in tissue mosaicism, and is not inherited through the germline. As a result of this mosaicism, the clinical disease spectrum of FD ranges from single bone involvement to multi-organ involvement. The most common cause is a missense mutation at either position c.602G>A (p.R201H) or c.601C>T (p.R201C). This mutation results in an amino acid substitution in the GTP hydrolase domain of the G_s_α protein, inhibiting the intrinsic GTPase activity, and leading to persistently elevated intracellular cAMP levels (Figure 1).

### Clinical Presentation of Fibrous Dysplasia

**Figure 2:**
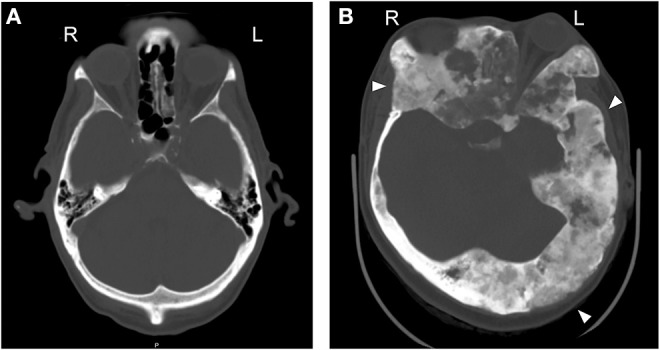
Fibrous dysplasia commonly affects the skull and is mosaic. Axial CT across the orbit. **(A)** A normal CT scan of the craniofacial bones, from a skeletally normal 61 y.o. male. **(B)** A 29 y.o. female with craniofacial FD. Note the asymmetry in the skull [ground glass expanded lesions (arrowheads) and displaced left globe].

A major clinical feature of MAS is FD of the bone, where expansile bone lesions cause fragility, malformations, and pain (Figure 2). FD also occurs without MAS, and is a common congenital skeletal dysplasia that can affect one bone (monoostotic) or multiple bones (polyostotic) (5). FD is arguably the most significant medical complication of MAS, since no effective treatments are available to manage the bone complications. In addition, the broad clinical spectrum of FD/MAS and the mosaic nature of the disease leads to variability in the radiographic presentation, making FD challenging to accurately diagnose.

In 2019, the FD/MAS International Consortium put forth a position statement to help guide clinicians in the diagnosis and management of FD/MAS (6). The first step in diagnosing FD/MAS is to perform a complete skeletal and extra-skeletal evaluation to determine the extent of the disease. MAS can be diagnosed if a patient has FD and at least one extraskeletal manifestation (café-au-lait skin hyperpigmentation, peripheral precocious puberty, thyroid lesions consistent with FD/MAS, growth hormone excess, neonatal hypercortisolism) or the absence of skeletal involvement but 2 extra-skeletal manifestations (6). An accurate diagnosis of FD/MAS can usually be made after a complete physical examination, combined with biochemical, hormonal, and radiologic evaluation of the skeletal, dermatologic, and endocrine systems. Biopsy of FD lesions is needed only if there is uncertainty about the diagnosis (i.e., atypical radiologic features) or concern for underlying malignancy (6). In these situations, affected tissue can be tested for the presence of a *GNAS* activating mutation, with the understanding that false negatives may occur if the sample has a low mutational burden. Peripheral blood is usually not sufficient for diagnosis due to the mosaicism of the disease. Next-generation sequencing is associated with a lower false-positive rate (6).

### Treatment and Monitoring of FD/MAS

Comprehensive guidelines regarding the management of the skeletal and extra-skeletal manifestations of FD/MAS were recently published, and should be considered when caring for patients along this clinical disease spectrum (6). The mainstay of therapy in FD/MAS remains adequate pain control, optimization of phosphate and vitamin D status, treatment of IGF-1 excess if present, and judicious consideration of surgical resection of FD lesions once they have stabilized. Unfortunately, there are no effective medical treatments available for FD/MAS. Bisphosphonate therapy in IV formulation has been reported to provide some benefits for pain control in patients with persistent moderate-to-severe pain from FD lesions, but why this helps in only some patients remains unclear (7–9). In addition, there is no evidence to suggest that bisphosphonates decrease the progression of FD lesions, and may not adequately control pain in some patients (7, 10).

Presently, there is minimal evidence for the use of denosumab and other anti-resorptive agents in FD, although there are case reports suggesting potential clinical benefits (8, 11–16). However, there are major concerns about rebound fractures and FD lesion progression after drug cessation (17–19). Ongoing clinical trials to address the utility of denosumab in FD are underway (NCT03571191). In addition, the TOCIDYS trial is evaluating the efficacy of IL-6 inhibition in patients with FD who did not have improvement in pain with prior bisphosphonate treatment (NCT01791842). These exciting trials hold promise for identifying potential medical strategies for mitigating the complications from FD.

## Mouse Models for Understanding FD

One contributor to the dearth of effective treatments for FD/MAS is the complexity of the *GNAS* locus. This complexity has made it challenging to develop robust mouse and human models to dissect the mechanisms of FD/MAS. During the past several years, novel strategies for uncovering the roles of G_s_-GPCR signaling in bone have been developed. These models provide critical insights into the pathogenesis and potential therapeutic approaches for FD.

One of the earliest models utilized the PTH/PTH-related protein (PTH/PTHrP) receptor (PPR), a GPCR, to study Jansen's metaphyseal chondrodysplasia (JMC). JMC is a rare form of short-limbed dwarfism caused by activating mutations of the PPR, leading to constitutive receptor activation and ligand-independent intracellular cAMP accumulation. Calvi et al. generated a mouse (Col1-caPPR) that expressed the human mutant PPR HKrk-H223R (caPPR), one of the causative mutations associated with JMC, in osteoblastic lineage cells in mice using a Col1 (2.3 kb) promoter (20). At 1 week of age, these mice showed increased osteoblast number and function in both the trabecular bone and the endosteal surface of cortical bone in the long bones. However, periosteal osteoblast activity was inhibited. This resulted in an increase in trabecular bone volume and a decrease in cortical bone thickness in the long bones. Calvarial thickness remained unchanged but there was increased porosity and bone remodeling on the endosteal surface of the skull. There was also an increased number of mature osteoclasts in these mice, which led to increased porosity of the cortical bone. At 2 weeks of age, excess bone formed in the bone marrow space (21). The area between the trabeculae was occupied by fibrous cells, blood vessels, and osteoclasts. There was delayed formation of bone marrow cavities, adipocytes, and hematopoietic cells. Surprisingly, these dysplastic bone and fibrous tissue phenotypes gradually resolved over time, and were limited to the metaphyseal area at 4 months. These studies showed that when the constitutively active PPR was expressed in osteoblastic lineage cells, the receptor could mediate both the anabolic and resorptive effects of PTH, and that PPR is involved in the regulation of both bone marrow and stromal tissues.

Our group used a different approach, creating an engineered GPCR RASSL (receptor activated solely by a synthetic ligand) to regulate GPCR signaling (22). The G_s_-coupled receptor, Rs1, was created by inserting a D100A mutation into the wild-type human 5HT4 serotonin receptor (23). This engineered receptor has a high basal level of constitutively active G_s_α activity and is not responsive to the endogenous serotonin ligand. The Col1(2.3)/Rs1 mouse model limits Rs1 expression spatially to osteoblastic lineage cells by using a Col1a1(2.3 kb) promotor, and temporally using the tet-off system, which allows for controlled expression of Rs1 in the absence of doxycycline. The FD-like phenotype of mice born off doxycycline was first apparent at 6 days (24). These mice showed age-dependent, increased trabecular bone formation with loss of marrow space and thinned cortical bone. The histologic and radiographic features strongly resemble human FD of the bone. There was also a dramatic increase in the number of immature osteoblasts present in the FD lesions, deposition of immature bone tissue, and reduced mineralization seen in analyses by FTIR spectroscopy and synchrotron radiation micro-computed tomography (25). The mice also showed lower numbers of hematopoietic stem cells (26). Col1(2.3)/Rs1 mouse bones showed dramatically reduced mature adipocyte differentiation, and higher osteoblastic glucose utilization than control mice (27). RNA analysis on whole bone samples showed increased Wnt signaling, suggesting that Wnt proteins may be a major driver of this effect (27). Importantly, blocking G_s_ signaling by using doxycycline at 4 weeks gradually reversed the bone phenotype (24), providing a proof-of-concept that therapies inhibiting G_s_ signaling may be effective for reversing fibrous dysplastic bone lesions. This model provides a powerful tool for understanding the effects of G_s_-GPCR signaling on dynamic bone growth and remodeling.

In contrast to strategies that did not directly manipulate *GNAS*, Saggio et al. generated a transgenic mouse model of human FD that constitutively expressed *GNAS-*R201C (28). The transgene expression did not show any appreciable effect on embryonic skeletal formation and unlike human FD, could be vertically transmitted. FD lesions were detected radiographically at 6 months of age. The lesions first appeared in the tail and later progressed to the femurs and the skull. The onset and progression of lesions were defined in three stages: an early phase (<2 months), defined by abnormal trabecular bone formation, endosteal thickening, and ectopic cortical lesions; an intermediate phase (2–6 months), with narrowed marrow cavity, expanded cortical bone and increased osteoclastic activity; and a late phase (>10 months) described as the fibrous dysplastic phase, with abnormal bone trabeculae and matrix fibrous tissue. At 1 year of age, the skeletal features resembled human FD.

When *GNAS*-R201C expression was limited to maturing osteoblasts using the Col1a1 (2.3 kb) promoter, changes in bone structure were detected on radiograph at 3 weeks in the tail, with excess bone mass (29). At 3 months, increased bone density was noted in all bones. The transgenic mice showed excess bone formation and remodeling of the bone marrow space. However, the expression in Col1a1(2.3 kb) cells failed to reproduce other features of human FD, such as bone marrow space fibrosis, and the loss of adipocytes and hematopoietic cells seen in other models (23, 28).

Palmisano et al. tested the effect of RANKL (receptor activator of nuclear factor kappa-B ligand) inhibition by treating the mice with an anti-RANKL antibody (30). They found that new and highly mineralized bone replaced the FD-like lesions in 2-month old mice, and the bone density increased. The treatment also stopped the growth of pre-existing FD lesions. In addition, there was a higher maximum load and stiffness of the bones in the treated group. However, the FD-like lesions progressed after RANKL inhibitor cessation.

More recently, Zhao et al. developed a mouse model expressing *GNAS*-R201C in the skeletal stem cell lineage using a tetracycline-inducible Cre-mediated Prrx1 driver (31). The FD bone phenotype was observed in embryos and adult mice <2 weeks after doxycycline administration (to activate the transgene) at E4.5. The FD lesions in the long bones showed reduced endochondral ossification, and in craniofacial bones showed decreased mineralization in the calvarial sutures. Poorly mineralized trabeculae and a dense fibrous matrix were present, and the bone marrow spaces were also decreased. When doxycycline was administered to 3-week old mice for 2 weeks (to induce expression), all limbs developed FD-like lesions with expansile bone deformities, fractures in limb bones, and defects in calvarial sutures. Doxycycline withdrawal resulted in reversal of the bone lesions. Hematopoietic cells and adipocytes appeared in the bone marrow of former FD lesions. The skulls of the reversed *GNAS*-R201C mice also showed normal morphology with healed sutures.

Finally, Khan et al. generated a new conditional knock-in mouse model [*GNAS*f(R201H)] using a minigene cassette to express *GNAS*-R201H, a human FD mutation, at the endogenous mouse *GNAS* locus (32). They found that expression of *GNAS*-R201H in the germ-line using Sox2-Cre resulted in embryonic lethality. Using Prrx1-Cre, they limited the expression of *GNAS*-R201H to osteochondral progenitor cells and early limb bud mesenchyme cells. At P0, control mice had well-formed cortical bone, cartilaginous epiphyses, and a normal marrow cavity. In contrast, Prrx1-Cre; *GNAS*f(R201H)/+ showed severely deficient long bone formation. At P6, Prrx1-Cre; *GNAS*f(R201H)/+ mice showed bone formation, but the marrow space was replaced by fibrous tissue and trabecular bone. In mutant long bones, higher levels of Wnt/β-catenin were also noted. At 3 weeks of age, there was more FD-like bone formation in the Prrx1-Cre; *GNAS*f(R201H)/+ mice; expansion of woven trabecular bone; lack of cortical bone; and increased osteoclasts. Furthermore, in this mouse model, Xu et al. demonstrated that G_s_α signaling mediated intramembranous ossification of cranial bones by regulating both Hh and Wnt/β-catenin signaling (33).

Together, these mouse models demonstrate that FD bone lesions can develop from activating mutations at the G_s_-GPCR level as well as from transgenic expression or knock-in expression of a *GNAS* allele carrying the R201C or R201H activating mutation. In addition, data from several mouse models suggest that blocking G_s_ pathway hyperactivity can lead to reversal of the FD bone lesions and may be a viable treatment strategy for human FD.

## Human Cellular Models of G_s_−Signaling

Human cellular models have contributed significantly to our understanding of FD/MAS pathophysiology, and patient-derived tissue samples have been an invaluable source. In 1998, Bianco et al. performed one of the earliest studies using FD/MAS patient bone marrow samples and showed that bone marrow stromal cell (BMSC) progenitors isolated from fibrous dysplastic lesions were either heterozygous for the G_s_α activating mutant allele, or homozygous wildtype (34). This discovery demonstrated that the skeletal progenitor cell population in FD lesions is mosaic, with a resident population of bone marrow stromal cells harboring the mutation and another population that is unaffected (34). When these cells were transplanted into immunocompromised mice, the wildtype colonies developed normal ossicles, but the colonies containing only the *GNAS* mutant allele did not survive and did not form ossicles. However, when a mixture of wildtype and mutant cell colonies was transplanted, an abnormal ossicle formed, with histopathologic features resembling FD. These experiments provided strong evidence that both wildtype and mutant cells were necessary to form a FD lesion, and that the mosaicism inherent to FD/MAS can be recapitulated within an FD lesion (34).

This novel concept of somatic mosaicism within an FD lesion was explored further by Kuznetsov et al. (35). In their study, they isolated colony forming unit-fibroblasts (CFU-Fs) from FD lesions of patients with FD/MAS and calculated the frequency of mutation-bearing CFU-Fs vs. normal CFU-Fs. They noted an inverse relationship between patient age and the number of mutated skeletal stem cell colonies present within an FD lesion, and concluded that the number of mutated stem cells must undergo apoptosis as patients age. Similarly, the bone histology in older patients (32–52 years of age) with FD/MAS was less severe than that of younger patients, and was more likely to be associated with a lower *GNAS* mutational burden. They hypothesized that as skeletal stem cells aged, there may be preferential apoptosis of mutated cells, resulting in loss of these cells and self-renewal of non-mutated cell populations. This could account for the clinically observed decreased incidence of new FD lesions and the relative stability of existing FD lesions as patients age. Kuznetsov et al. also transplanted cell colonies containing CFU-Fs into immunocompromised mice and showed that FD ossicles formed, whereas non-mutant CFU-Fs or mutation-positive strains without mutant skeletal stem cell populations did not form FD ossicles. Thus, they concluded that *GNAS* mutations within the skeletal stem cell population was sufficient to induce formation of FD lesions (35).

Piersanti et al. developed a model in which human skeletal progenitor cells were engineered to stably over-express the *GNAS*-R201C mutation using a lentiviral vector (36). These cells demonstrated elevated cAMP production, consistent with over-expression of G_s_α. When cultured *in vitro*, they did not exhibit mineralization and had lower osteocalcin levels than controls. Levels of the osteogenic markers alkaline phosphatase and bone sialoprotein were elevated compared to controls, and the cells exhibited robust RANKL expression, consistent with the profound osteoclastogenesis seen in most human FD lesions. Additionally, genes in the phosphodiesterase pathway were upregulated in these cells, suggesting an adaptive response to G_s_α over-expression. When these cells were transplanted into immunocompromised mice, the stably-transduced *GNAS*-R201C cells formed ossicles but were unable to differentiate into adipocytes or hematopoietic components. Finally, silencing of the *GNAS*-R201C allele with lentiviral vectors containing short hairpin interfering RNA sequences caused these cells to revert to their normal state and no longer exhibit a mutant phenotype.

In 2019, de Castro et al. used human FD-derived BMSCs from a well-characterized cohort of FD patients at the NIH to show that RANKL expression in FD skeletal lesions may directly contribute to osteoclast induction in FD lesions (37). They showed that serum levels of RANKL were 16-fold higher in FD patients compared with healthy controls, and the serum RANKL/OPG ratio was 12-fold higher. The magnitude of increase in RANKL and RANKL/OPG was positively correlated with total body skeletal disease burden score, a well-validated scoring system used to determine the severity of FD.

de Castro et al. also isolated BMSCs from these FD patients and healthy volunteers and showed that RANKL levels were higher in the conditioned media of FD BMSCs compared with BMSCs derived from healthy volunteers when stimulated with prostaglandin E2 (PGE2) and 1,25 vitamin D3 (37). These cells also released OPG, but to a much lower degree than healthy volunteer controls. Additionally, when FD BMSCs were co-cultured in osteogenic media with peripheral monocytes from healthy volunteers, the monocytes differentiated into TRAP+ osteoclasts; however, when cultured in non-osteogenic media, they did not induce osteoclastogenesis. They subsequently showed that osteoclastogenesis could be inhibited when the cell co-cultures were treated with denosumab. This study provided strong evidence that RANKL is expressed in human FD lesions and is correlated with disease burden, thus implicating RANKL over-expression as an important contributor to FD pathogenesis and informing the design of ongoing studies testing denosumab in FD (NCT03571191).

## Conclusions

The past 15 years have shown major advances in our understanding of FD/MAS. There are striking consistencies among the mouse models of FD, and a number of features of the human disease are replicated in the mouse genetic models, suggesting that direct targeting of the G_s_ pathway has strong potential as a therapeutic strategy for FD. In addition, the human cell models of FD are providing new tools for understanding FD pathogenesis and cell-type specific effects of the *GNAS* activating mutations. Finally, international collaborations among clinicians and researchers with strong experience in FD/MAS are yielding best-practice recommendations and treatment guidelines for optimal management of FD/MAS.

## Author Contributions

EH, KW, and HL conceived of this manuscript, wrote the manuscript, and edited the manuscript.

### Conflict of Interest

EH and KW receive clinical trial research support through their institution from Clementia Pharmaceuticals for clinical trials unrelated to this work. EH is also a volunteer on the editorial board for the Journal of Bone and Mineral Research. EH serves in an unpaid capacity on the International FOP Association Medical Registry Advisory Board, on the International Clinical Council on FOP, and on the Fibrous Dysplasia Foundation Medical Advisory Board. EH has also received clinical trial research support through his institution from Regeneron Pharmaceuticals. The remaining author declares that the research was conducted in the absence of any commercial or financial relationships that could be construed as a potential conflict of interest.
